# A Social Network Analysis of Hemodialysis Clinics: Attitudes Toward Living Donor Kidney Transplant among Influential Patients

**DOI:** 10.34067/KID.0000000000000383

**Published:** 2024-02-07

**Authors:** Hannah Calvelli, Heather Gardiner, Crystal Gadegbeku, Peter Reese, Zoran Obradovic, Edward Fink, Avrum Gillespie

**Affiliations:** 1 Lewis Katz School of Medicine at Temple University, Philadelphia, Pennsylvania; 2 Temple University College of Public Health, Philadelphia, Pennsylvania; 3 Cleveland Clinic Glickman Urological and Kidney Institute, Cleveland, Ohio; 4 Department of Medicine, University of Pennsylvania, Philadelphia, Pennsylvania; 5 Temple University Center for Data Analytics and Biomedical Informatics, Philadelphia, Pennsylvania; 6 Temple University School of Media and Communication, Philadelphia, Pennsylvania; 7 Temple University Hospital, Philadelphia, Pennsylvania

**Keywords:** CKD, community engagement and health, hemodialysis, kidney failure, kidney transplantation, outcomes, social determinants of health, transplantation

## Abstract

**Key Points:**

- Hemodialysis clinic social networks spread attitudes and behaviors toward kidney transplants.
- Identifying and characterizing influential patients is a first step in future hemodialysis clinic social network interventions to promote kidney transplantation.

**Background:**

Hemodialysis clinics help develop patient social networks that may spread kidney transplant (KT) attitudes and behaviors. Identifying influential social network members is an important first step to increase KT rates. We mapped the social networks of two hemodialysis facilities to identify which patients were influential using in-degree centrality as a proxy for popularity and influence.

**Methods:**

In this cross-sectional study, we performed a sociocentric social network analysis of patients on hemodialysis in two geographically and demographically different hemodialysis facilities. Statistical and social network analyses were performed using R statistical software.

**Results:**

More patients at facility 1 (*N*=71) were waitlisted/evaluating living donor KT (50.7% versus 20.0%, *P =* 0.021), considered KT as very important (70.4% versus 45.0%, *P =* 0.019), and knew people who received a successful KT (1.0 versus 0.0, *P =* 0.003). Variables predicting relationship formation at facility 1 were the same shift (*β*=1.87, 95% confidence interval [CI] [1.19 to 2.55]; *P <* 0.0001), same sex (*β*=0.51, 95% CI [0.01 to 1.00]; *P =* 0.045), younger age (*β*=−0.03, 95% CI [−0.05 to −0.01]; *P =* 0.004), different lengths of time on hemodialysis (*β*=−0.49, 95% CI [−0.86 to −0.12]; *P =* 0.009), and knowing more people who received a successful KT (*β*=0.12, 95% CI [0.03 to 0.21]; *P =* 0.009). Predictive variables at facility 2 (*N*=40) were the same race (*β*=2.52, 95% CI [0.39 to 4.65]; *P =* 0.021) and knowing fewer people with successful KT (*β*=−0.92, 95% CI [−1.82 to −0.02]; *P =* 0.045). In-degree centrality was higher at facility 1 (1.1±1.2) compared with facility 2 (0.6±0.9).

**Conclusions:**

Social networks differed between the hemodialysis clinics in structure and prevalent transplant attitudes. Influential patients at facility 1 (measured by in-degree centrality) had positive attitudes toward KT, whereas influential patients at facility 2 had negative attitudes.

## Visual Abstract

**Figure:**
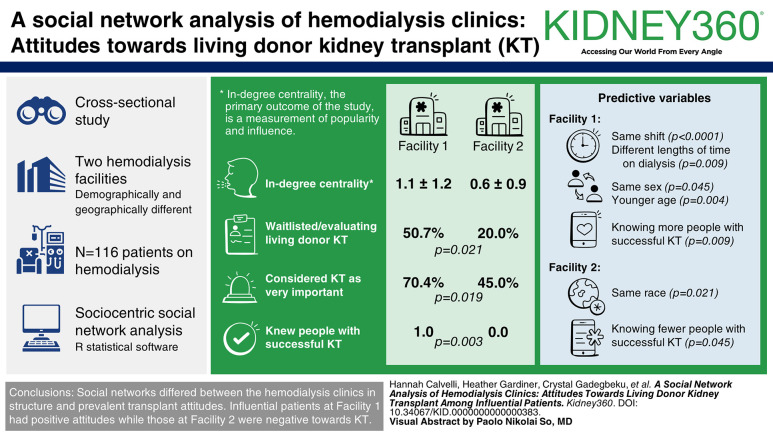

## Introduction

### Background/Rationale

Among patients with ESKD, kidney transplantation provides the best possible outcomes and quality of life. Kidney transplantation is underutilized in the United States, especially among underserved populations. This represents a significant health disparity with multiple barriers to receiving a kidney transplant (KT), including a lack of knowledge about transplantation.^1^ As a result, in-center hemodialysis is the dominant treatment modality for patients with ESKD. Interventions are needed in the hemodialysis clinic to improve access to transplant-related information among patients with ESKD.

Social network interventions present a potential means for increasing transplant knowledge. Hemodialysis clinics are unique environments that facilitate the formation of social networks among patients with ESKD, as patients spend an average of 12 hours on hemodialysis per week in a group setting amenable to the formation of social ties.^2^ Social network theory posits that the dissemination and transmission of information within social networks can catalyze changes in attitudes and behaviors among network members. This has been previously demonstrated by Christakis and Fowler, who found that behaviors leading to obesity and smoking are spread through social ties.^3,4^

Among patients with ESKD, social network analysis (SNA) has shown that those who receive factual information about transplantation from social network members are more likely to be evaluated for a KT.^5,6^ In developing targeted interventions that leverage patient social networks, it is important to understand how the hemodialysis clinic social network facilitates the spread of transplant-related information and, in turn, influences patient attitudes and behaviors toward transplantation.^7,8^

Implementing targeted social network interventions requires first identifying who is most popular and influential within the network. This can be accomplished by analyzing centrality, a measure of how and how much network members are connected to each other.^8^ In this study, we explored in-degree centrality as a proxy for popularity and influence within the social network. In-degree centrality represents the number of incoming relationships a given network participant has, and recent studies have been published on how in-degree centrality contributes to patient attitudes and behaviors.^9^ Ours is the first study focused on in-degree centrality within hemodialysis clinic social networks.^10,11^

### Objectives

Our primary objective was to conduct a SNA of in-degree centrality at two hemodialysis clinics. We also explored the structural, demographic, clinical, and attitudinal differences between the patients. We hypothesized that higher in-degree centrality was associated with prevailing attitudes within the hemodialysis clinic social network.

## Methods

### Study Design

This study was the baseline cross-sectional analysis of Social Networks and Renal Education: Promoting Transplantation, NCT03536858. Data were collected as part of a social network intervention clinical trial of in-center hemodialysis. The Temple University Institutional Review Board approved the study protocol. Written informed consent was obtained. The clinical and research activities being reported are consistent with the Principles of the Declaration of Istanbul, as outlined in the “Declaration of Istanbul on Organ Trafficking and Transplant Tourism” as well as the Declaration of Helsinki.

### Setting

This survey was designed to be a census of our two hemodialysis facilities (facility 1 in southeastern Pennsylvania and facility 2 in central New Jersey). These clinics were selected because they were part of the same dialysis organization but demographically and geographically different.

### Participants

A roster of patients receiving hemodialysis was generated at the beginning of the recruitment period. Patients were eligible to participate if they had ESKD, spoke English, and were 18 years or older. Patients were approached to participate during their hemodialysis sessions. As previously described, patients were excluded if they declined to participate, could not consent (*e.g*., cognitive, severe visual, or hearing impairment), were hospitalized, switched to peritoneal dialysis, received a transplant, transferred out, died before they could be surveyed, or were asleep during the recruitment period (Supplemental Figure 1).^12^ Participants received a $10 gift card on completion of the survey.

### Variables

Demographic variables included dialysis shift, age, sex, race, education, employment, income, marital status, and religion. Dialysis shift was Monday/Wednesday/Friday or Tuesday/Thursday/Saturday. Age was treated as a continuous variable, and all other demographic variables were treated as categorical. Clinical variables included self-reported health status, medical conditions, highest stage in the transplant evaluation process, time on hemodialysis, number of known patients with successful versus unsuccessful KTs, level of trust in doctors, importance of a KT, and willingness to accept a living versus deceased donor KT. These variables were selected because they were previously shown to be associated with the likelihood of receiving a KT.^13–15^ Self-reported health status, medical conditions, highest stage, and time on hemodialysis were treated as categorical variables, and all other clinical variables were treated as continuous.

### Outcomes

Our primary outcome of interest was in-degree relationship formation, as patients with higher in-degree centrality are more likely to form in-degree relationships.

### Data Sources/Measurement

Research electronic data capture was used for questionnaire administration and data management. We used an interviewer-administered computer-based questionnaire for data collection. The questionnaire, which combined three previously validated surveys, was designed to identify and quantify the relationships within the social networks of patients on hemodialysis.^13,16^ The questionnaire also assessed participants' attitudes regarding kidney disease as well as demographic and clinical variables.

The social network questionnaire used three questions to identify members of patients' social networks: (*1*) whom are the patients you talk to? (*2*) whom are the patients you discuss kidney disease with? and (*3*) whom are the patients you discuss KTs with? To avoid recall bias, participants could identify up to 12 patients, which approaches the limit of accurate recall while minimizing cognitive burden.^17^

To assess patients' knowledge about people receiving KTs, participants were asked, “Including yourself, how many people do you know who had a successful KT?” They were also asked, “How many people do you know who had a KT but had to go back on dialysis or died soon after surgery?” Attitude toward KT was measured by a survey question that asked, “In your opinion, how important is it for you to get a KT?”

Transplant eligibility and steps toward transplantation was self-reported using a schema developed by Sullivan *et al.* in 2012: (*1*) suitability for referral to transplant center, (*2*) interest in transplantation, (*3*) referral call to transplant center, (*4*) first visit to transplant center, (*5*) transplant center workup, (*6*) workup complete, (*7*) active on the list, and (*8*) successfully received a KT.^18^ Participants' surveys were excluded from these analyses if sections of the questionnaire were unanswered or if the survey was <90% complete.

### Statistical Tests

Chi-squared and Fisher's exact tests were used to test the statistical significance of independent demographic and clinical variables' associations with categorical dependent variables. ANOVA was used for continuous variables. All tests were two-tailed with *P <* 0.05 considered statistically significant. Categorical and dichotomous variables are presented as frequency counts and percentages. Continuous variables are summarized by their mean and SD or median and interquartile range.

### Network Statistics

Survey participants who spoke with other survey participants were defined as part of the hemodialysis clinic patient social network. All identified relationships were compiled into an adjacency matrix to calculate network statistics and visualizations. This resulted in a directed network graph for each clinic.

### SNA

Exponential random graph models (ERGMs) were generated to examine the influence of multiple covariates on in-degree relationship formation while controlling for network structural features.^19^ We assessed how both sociality (the likelihood of forming a relationship) and homophily (the likelihood of forming a relationship with someone of the same level of a categorical attribute or same value of a continuous attribute) contributed to in-degree centrality for the following demographic and clinical variables: dialysis shift, age, race, sex, time on hemodialysis, and number of known successful KTs. The transitivity term was included to determine the inherent tendency for triadic closure (the likelihood that a friend of a friend is also your friend). The mutual term was included as a measure of reciprocity (the likelihood that someone you identify as a friend also identifies you as their friend) (Box 1).

Box 1 **Glossary of terms**

**Table:** 

Term	Definition
**Social network theories**	
Centrality	The tendency for a few members to have many links while most other members have one or two links. (Example: if member A joins the network and member B has five links and member C has one link, member A would preferentially form a link with member B.)
Sociality	The tendency for people to form a relationship regardless of the other member's characteristics
Homophily	The tendency for people to form a relationship based on similar characteristics of the other member
Transitivity	The tendency for network members to share relationships
Triad closure	The tendency for network members to form relationships with friends of a friend. (Example: if member A is linked to member B and member C, it is likely that members B and C are also linked.)
Mutuality	The tendency for network members to form reciprocal relationships. (Example: if member A is linked to member B, it is likely that member B is also linked to member A)
**Social network analysis**	
Nodes	A social network term for network members
Links	A social network term for relationships between two network members
Edges	Another term for a link or relationship used in graph theories
Degree	The number of relationships (links) a network member has
In-degree	The number of relationships (links) directed toward a network member
Exponential random graph model (ERGM)	ERGM analyzes the network as a multivariate observation with a link as the dependent variable. The observed network is then compared to 100,000 randomly generated Markov random graphs (networks) using maximum pseudolikelihood estimation and Monte Carlo maximum likelihood estimates (MC MLE)
Geometrically weighted in-degree	Geometrically weighted in-degree weights the probability of a network member forming a relationship based on the number of other members who preferentially form relationships with them (in-degree centrality). This parameter is used in ERGMs to approximate centrality within the network
Geometrically weighted edgewise shared partner	Geometrically weighted edgewise shared partner weights the probability of two members forming a relationship based on the number of relationships with other members they have in common. This parameter is used in ERGMs to approximate clustering within the network
Incoming shared partner (ISP)	ISP. (Example: vertex *k* is an ISP of ordered pair (*i*,*j*) iff *k*→*i*,*k*→*j*)
Akaike information criterion	Akaike information criterion. Estimates how well a model fits the data it was generated from
Markov chain Monte Carlo	A stochastic algorithm used to approximate maximum likelihood estimates for ERGMs
Monte Carlo maximum likelihood estimate	An approximate maximum likelihood estimate based on a Monte Carlo scheme
Goodness of fit	A function that calculates *P* values for geodesic distance, degree, and reachability summaries for exponential family random graph models
Marginal effects plot	Computes predicted values for all possible levels and values from a model's predictors

The geometrically weighted in-degree modeled centralization and the directed geometrically weighted edgewise shared partner (GWESP) modeled clustering.^20^ We further specified the GWESP shared partner type as incoming shared partner, which best measured multiple triadic closure and in-degree.^21^ Geometrically weighted in-degree and GWESP have decay parameters between 0 and 1, and they were optimized to the lowest Akaike information criterion for the most parsimonious model.^20^

The observed network was compared with 100,000 random graphs using maximum pseudolikelihood estimation and Monte Carlo Maximum Likelihood Estimates.^22^ Models were optimized based on Goodness of Fit and Markov Chain Monte Carlo diagnostics (Supplemental Figures 2 and 3). See Supplemental Table 1 for the full unoptimized ERGM models. Marginal Effects Plots were generated for variables that were significantly associated with in-degree relationship formation in the ERGM, to visualize the probability of relationship formation along the full range of the variable (*e.g*., from knowing no successful KTs to knowing ten or more successful KTs).

### Software

Descriptive statistics, SNA, and exponential random graph modeling were performed with R statistical software, version 4.2.2.^23^

## Results

### Demographic Variables

Between October 2018 and October 2020, 116 patients were enrolled; however, five were excluded for incomplete surveys. The response rates were similar between facility 1 and facility 2 (57% and 53%), with no differences in age or sex characteristics between social network participants and the total number of patients at both facilities (Supplemental Figure 4). Table 1 presents the demographic variables for the two facilities. Of the 111 patients analyzed in this study, 71 (64.0%) were from facility 1 in southeastern PA and 40 (36. 0%) from facility 2 in central NJ. Patients differed by race (*P <* 0.001), with more patients at facility 1 who were African American compared with facility 2 (91.5% versus 40.0%). Fewer patients at facility 1 attended college compared with facility 2 (43.7% versus 65.0% *P =* 0.035).

**Table 1:** Patient demographic variables by facility

Variable	Facility 1 (*N*=71)	Facility 2 (*N*=40)	*P* Value
**Age, yr**			
Mean (SD)	58.5 (12.9)	63.0 (12.7)	0.076
Median (min, max)	60.0 (31.0, 85.0)	63.5 (32.0, 89.0)	
**Sex, *n* (%)**			
Male	35 (49.3)	26 (65.0)	0.16
Female	36 (50.7)	14 (35.0)	
**Race, *n* (%)**			
African American	65 (91.5)	16 (40.0)	<0.001
Non–African American	6 (8.5)	24 (60.0)	
**Hispanic or Latino, *n* (%)**			
Yes	3 (4.2)	3 (7.5)	0.77
**Attended college, *n* (%)**			
Yes	31 (43.7)	26 (65.0)	0.035
Missing		1 (2.5)	
**Employed, *n* (%)**			
Yes	4 (5.6)	4 (10.0)	0.64
**Yearly income (dollars), *n* (%)**			
Under 20,000	42 (59.2)	21 (52.5)	0.63
Over 20,000	29 (40.8)	19 (47.5)	
**Married, *n* (%)**			
Yes	18 (25.4)	18 (45.0)	0.056
**Religious, *n* (%)**			
Yes	66 (93.0)	34 (85.0)	0.31
**Frequency of religious service attendance, *n* (%)**			
More than once a month	26 (36.6)	21 (52.5)	0.15
Less than once a month	45 (63.4)	19 (47.5)	

*P* value < 0.05 was considered as significant.

### Clinical Variables

Table 2 presents the clinical variables for the two facilities. Facility 1 had a greater proportion of patients who were on the KT waitlist or being evaluated for living donor KT compared with facility 2 (50.7% versus 20.0%, *P =* 0.021). Furthermore, patients at facility 1 knew more people who had received a successful KT compared with facility 2 (median 1.0 versus 0.0 people, *P =* 0.003). A greater proportion of patients at facility 1 considered KT very important compared with facility 2 (70.4% versus 45.0%, *P =* 0.019).

**Table 2:** Patient clinical variables by facility

Variable	Facility 1 (*N*=71)	Facility 2 (*N*=40)	*P* Value
**Health status, *n* (%)**			
Very good	8 (11.3)	8 (20.0)	0.62
Good	23 (32.4)	12 (30.0)	
Fair	32 (45.1)	15 (37.5)	
Poor	8 (11.3)	5 (12.5)	
**Cancer, *n* (%)**			
Yes	15 (21.1)	3 (7.5)	0.11
**Heart disease, *n* (%)**			
Yes	13 (18.3)	2 (5.0)	0.093
**Peripheral vascular disease, *n* (%)**			
Yes	7 (9.9)	2 (5.0)	0.59
**Lung disease, *n* (%)**			
Yes	9 (12.7)	3 (7.5)	0.60
**On waitlist or evaluating transplant, *n* (%)**			
Yes	36 (50.7)	8 (20.0)	0.021
Missing	3 (4.2)	9 (22.5)	
**Number known successful KT**			
Mean (SD)	2.50 (4.39)	0.775 (1.14)	0.0026
Median (min, max)	1.00 (0, 30.0)	0 (0, 4.00)	
**Number known unsuccessful KT**			
Mean (SD)	1.17 (2.68)	0.650 (1.19)	0.16
Median (min, max)	0 (0, 20.0)	0 (0, 6.00)	
**Trust doctors, *n* (%)**			
Not a lot	33 (46.5)	20 (50.0)	0.93
A lot	37 (52.1)	20 (50.0)	
Missing	1 (1.4)		
**Importance of KT, *n* (%)**			
Not at all	9 (12.7)	12 (30.0)	0.019
Somewhat	11 (15.5)	10 (25.0)	
Very	50 (70.4)	18 (45.0)	
Missing	1 (1.4)		
**Accept LDKT, *n* (%)**			
Yes	60 (84.5)	30 (75.0)	0.25
**Accept DDKT, *n* (%)**			
Yes	61 (85.9)	33 (82.5)	0.70

DDKT, deceased donor kidney transplant; KT, kidney transplant; LDKT, living donor kidney transplant.

Health status was self-reported by patients. Number known was reported as the number of people identified by the patient with either a successful or unsuccessful kidney transplant. *P* value < 0.05 was considered as significant.

### In-Degree Centrality

**Figure 1:**
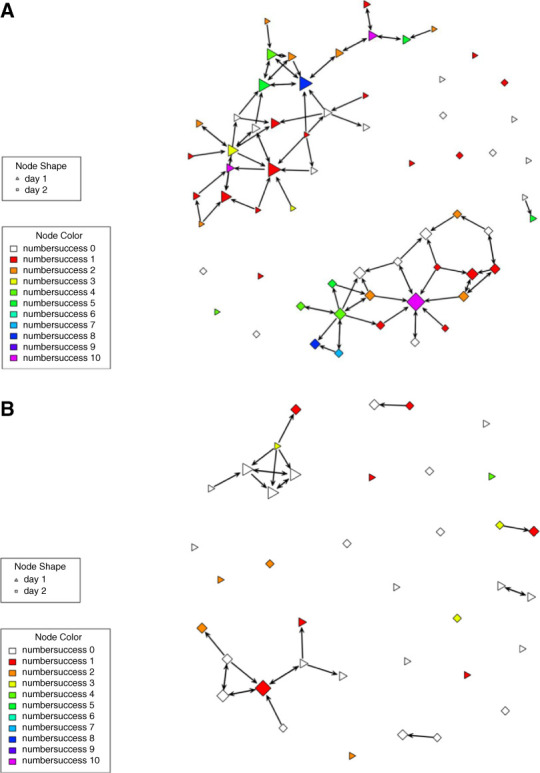
**Network plot by facility**. (A) Facility 1. (B) Facility 2. The shapes (nodes) represent participants in the hemodialysis facility social network. The black lines (edges) represent a relationship with the arrows indicating directionality. Node shape represents dialysis day either day 1 (Monday/Wednesday/Friday) or day 2 (Tuesday/Thursday/Saturday). Node color represents how many people the participant knew who received a successful KT. Node size represents in-degree centrality where larger nodes had higher in-degree centrality. KT, kidney transplant.

**Figure 2:**
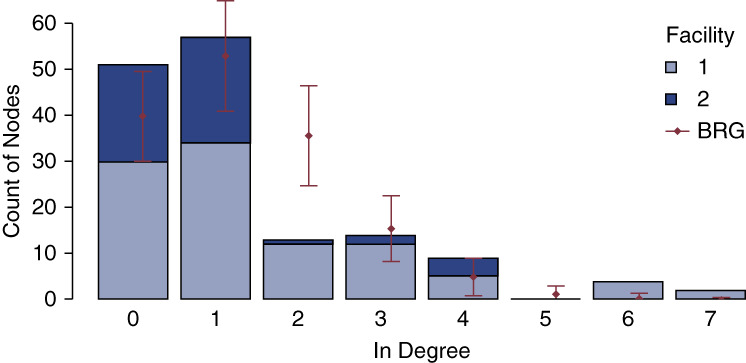
**In-degree centrality distribution graph by facility**. Histogram showing the distribution of in-degree centrality by facility. In-degree centrality is represented on the *x* axis, and frequency is represented on the *y* axis. BRG displays the null model. BRG, Bernoulli random graph.

Figure 1 shows the social networks maps for the two facilities. Patients at facility 1 had higher in-degree centrality and knew more people who had received a successful KT compared with facility 2. Figure 2 shows the distribution of in-degree centrality at both facilities. Facility 1 had higher in-degree centrality compared with facility 2 (1.5±1.7 relationships versus 0.9±1.2 relationships, *P <* 0.001).

### Variables Associated with In-Degree Centrality

Table 3 presents the ERGMs for facility 1 and facility 2, which predict the probability of in-degree relationship formation and the variables associated with in-degree centrality. We first assessed the inherent likelihood of relationship formation (Edges) as well as the likelihood of reciprocating the relationship once it is formed (Mutual) before adding variables associated with in-degree centrality. Edges and Mutuals are interpreted as the intercept of the model. At facility 1, the model had a low inherent likelihood of patients forming a relationship in the absence of any variables (*β*=−4.32, 95% confidence interval [CI] [−6.35 to −2.29]; *P <* 0.0001); however, when they did, there was a high likelihood of reciprocity among relationships formed (*β*=2.89, 95% CI [2.11 to 3.67]; *P <* 0.0001).

**Table 3:** Exponential random graph model by facility

Variables	Facility 1	Facility 2
**Structural variables, *β* (SEM)**		
Edges	−4.36 (1.04)^a^	−5.10 (2.98)
Mutual	2.94 (0.41)^a^	1.69 (0.99)
GWIDEGREE (0.5)	−0.81 (0.48)	
GWESP ISP (0.1)	0.32 (0.19)	
GWIDEGREE (0.1)		1.40 (1.06)
GWESP ISP (0)		−0.49 (0.86)
**Demographic variables, *β* (SEM)**		
Shift (sociality), REF=AM	0.33 (0.19)	−0.42 (0.71)
Shift (homophily)	1.87 (0.35)^a^	0.53 (0.46)
Age (sociality)	−0.03 (0.01)^b^	−0.03 (0.03)
Age (homophily)	−0.02 (0.01)	−0.05 (0.03)
Race (sociality), REF=African American		−1.56 (0.87)
Race (homophily)		2.54 (1.07)^c^
Sex (sociality), REF = Male	0.59 (0.23)^c^	0.24 (0.66)
Sex (homophily)	0.51 (0.26)^c^	0.17 (0.60)
Transitivity (sex)	−0.51 (0.32)	1.67 (0.86)
**Clinical variables, *β* (SEM)**		
Time on hemodialysis (sociality)	0.25 (0.23)	0.54 (0.56)
Time on hemodialysis (homophily)	−0.49 (0.18)^b^	−0.30 (0.42)
Number known successful KT (sociality)	0.13 (0.05)^b^	−0.92 (0.47)^c^
Number known successful KT (homophily)	−0.02 (0.04)	−0.06 (0.24)
**Model factors**		
AIC	545.53	151.69
BIC	629.02	228.71
Log likelihood	−257.76	−58.84

AIC, Akaike's Information Criterion; BIC, Bayesian information criterion; GWESP, geometrically weighted edgewise shared partner; GWIDEGREE, geometrically weighted in-degree; ISP, incoming shared partner; KT, kidney transplant; REF, reference.

a *P* value < 0.001.

b *P* value < 0.01.

c *P* value < 0.05.

**Figure 3:**
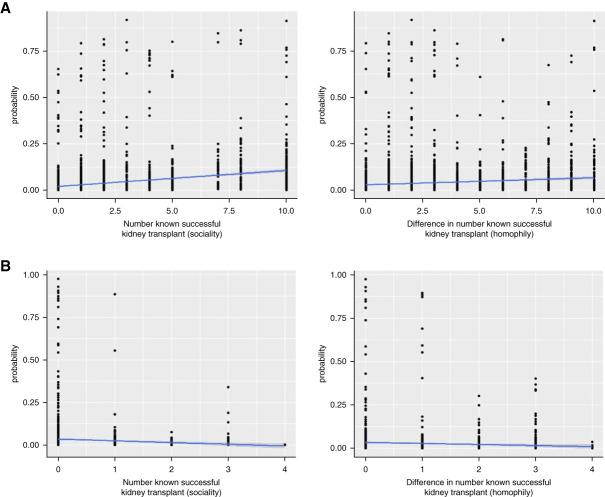
**Marginal effects plot number known successful transplant by facility**. (A) Facility 1. (B) Facility 2. Plot showing the marginal effects of number of known successful KTs (sociality) or the absolute difference in the number of known successful KTs (homophily) (*x* axis) on probability of tie formation (*y* axis). Sociality=likelihood of forming a relationship on the basis of the number of known successful KTs (*e.g*., if you know of many successful KTs, you are more likely to form many relationships). Homophily=likelihood of forming a relationship with someone who knows the same number of successful KTs (*e.g*., if you do not know any people who have had a successful KT, you are more likely to form relationships with other participants who do not know of any successful KTs).

We next added demographic and clinical variables to the model to determine their association with in-degree centrality. At facility 1, participants with higher in-degree centrality were younger (*β*=−0.03, 95% CI [−0.05 to −0.01]; *P =* 0.004) and female (*β*=0.57, 95% CI [0.10 to 1.04]; *P =* 0.017). Participants who knew more successful KT recipients had higher in-degree centrality with the likelihood increasing with each additional successful transplant recipient they knew (*β*=0.12, 95% CI [0.03 to 0.21]; *P =* 0.009). Figure 3 is a Marginal Effects Plot to visualize the associations between in-degree centrality and the number of known successful KTs on the basis of sociality and homophily. At facility 1, there was a 10% probability of in-degree relationship formation for participants who knew ≥10 successful KTs compared with 3% for participants who knew no successful KTs. There was no significant homophily effect for knowing successful KTs, but there was an increased probability of in-degree relationship formation among those who knew different numbers of successful KTs compared with the same number (7% versus 4%, respectively).

**Figure 4:**
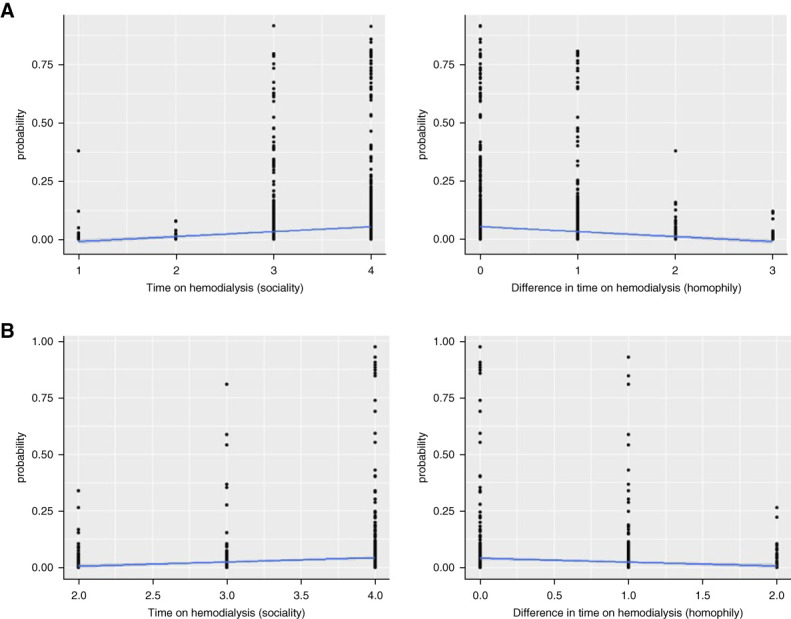
**Marginal effects plot time on hemodialysis by facility**. (A) Facility 1. (B) Facility 2. Plot showing the marginal effects of time on hemodialysis (sociality) or the absolute difference in time on hemodialysis (homophily) (*x* axis) on probability of tie formation (*y* axis). Sociality=likelihood of forming a relationship on the basis of the amount of time spent on hemodialysis (*e.g*., if you have spent a longer time on hemodialysis, you are more likely to form many relationships). Homophily=likelihood of forming a relationship with someone who has spent the same amount of time on dialysis (*e.g*., if you have spent less than 6 months on dialysis, you are more likely to form relationships with other participants who have spent less than 6 months on dialysis).

At facility 1, there was homophily effect for participants to form relationships if they were on the same shift (*β*=1.87, 95% CI [1.19 to 2.55]; *P <* 0.0001) and of the same sex (*β*=0.51, 95% CI [0.01 to 1.00]; *P =* 0.045). Participants formed relationships with people of different ages (*β*=−0.02, 95% CI [−0.04 to 0.00]; *P =* 0.047) and with people who were on hemodialysis for similar lengths of time (*β*=−0.49, 95% CI [−0.86 to −0.12]; *P =* 0.009). As shown in Figure 4, there was an approximate 6% probability of relationship formation for participants on hemodialysis for >5 years compared with <1% for participants on hemodialysis for <6 months. Through the homophily effect, there was an increased probability of relationship formation among those on hemodialysis for the same amount of time compared with different amounts of time (6% versus <1%, respectively).

At facility 2, there was also a low baseline tendency to form a relationship (*β*=−5.20, 95% CI [−8.22 to −2.19]; *P =* 0.085) and a positive but not a significant probability of reciprocating the in-degree relationship (*β*=1.71, 95% CI [0.72 to 2.70]; *P =* 0.083). In contrast to facility 1, at facility 2, knowing fewer successful KT recipients (*β*=−0.92, 95% CI [−1.82 to −0.02]; *P =* 0.045) was associated with higher in-degree centrality (Table 2). As shown in the Marginal Effects Plot in Figure 3, for sociality, there was a <1% probability of relationship formation for participants who knew ≥4 successful KTs compared with 4% for participants who knew no successful KTs. Other factors associated with in-degree centrality at facility 2 were race, sex, and time on hemodialysis. Participants were more likely to form relationships if they were of the same race (*β*=2.52, 95% CI [0.39 to 4.65]; *P =* 0.021). Participants of the same sex tended to form relationships in groups of three (triadic closure, *β*=1.62, 95% CI [0.00 to 3.24]; *P =* 0.049). Participants who were on hemodialysis longer had higher in-degree centrality (Table 3), which was further characterized using a Marginal Effects Plot (Figure 4). The Marginal Effects Plot for sociality demonstrated a 5% probability of relationship formation for participants on hemodialysis for >5 years compared with <1% for participants on hemodialysis for <6 months. For homophily, there was an increased probability of relationship formation among those on hemodialysis for the same amount of time compared with different amounts of time (5% versus <1%).

## Discussion

In this SNA of people on hemodialysis, we characterized structural, attitudinal, and demographic variables at two facilities. Participants at facility 1 had higher in-degree centrality compared with facility 2. Participants at facility 1 also knew more people who had received a successful KT. This difference in knowledge surrounding KTs is an important finding given that both positive and negative health attitudes and behaviors can disseminate through interpersonal relationships, allowing one person's behavior to cascade across entire networks.^24,25^ For example, the popular patients at facility 1, who knew of more successful KTs, may have more positive attitudes toward KT and promote transplantation through their social network. Conversely, at facility 2, the popular patients knew of fewer successful KTs and may spread negative attitudes toward KT through their network. This idea is further supported by our finding that patients with high in-degree centrality spent a longer time on hemodialysis; thus, they may be perceived by others in the social network as more experienced or knowledgeable about KT.

Many barriers to KT are exacerbated by the social determinants of health (SDOH). SDOH have become widely recognized for their associations with a multitude of health outcomes across diverse settings and populations.^26^ A study by Wesselman *et al.* in 2021 found that African American patients had a lower likelihood of receiving a KT and longer wait times to transplantation.^27^ This is likely multifactorial due to delays in completing the evaluation process and a relative lack of suitable matched organs for living donor KT.^28^ A 2022 study by Iroegbu *et al.* demonstrated that patients of minority races had less awareness about the importance of ESKD management.^29^ Furthermore, a 2021 study by Ng *et al.* showed that perceived racism and medical mistrust were more prevalent among African American patients and that they had less KT knowledge compared with White patients.^30^

In our study, there was no significant difference in how much patients trusted doctors at the two facilities, further suggesting that factors besides race, such as in-degree centrality and other social network factors, contributed to the differing attitudes and behaviors of the two facilities. Our findings raise questions about why these disparities in ESKD persist and how they may be affected by social network structures. The facilities in our study differed by race, income (greater than or less than the federal poverty level of $20,000 for a household of two), and education.^31^ However, patients at facility 1, who were predominantly self-identified as Black and reported less education, had positive attitudes, knowledge, and behaviors toward kidney transplantation, as well as higher in-degree centrality. Thus, it is possible that this hemodialysis network provided resilience against the SDOH.

Given the demonstrated influence of social network structures within our patient cohort and the advances in SNA, our findings suggest a role for future interventions to improve transplantation rates and address disparities in kidney health.^32^ For example, a potential network intervention could include (*1*) identifying patients with high-indegree centrality through social network mapping, (*2*) educating patients with high in-degree centrality about the risks versus benefits of KT, (*3*) training these patients on how to share this information in a conversational manner, and (*4*) encouraging them to participate in conversations regarding KT during hemodialysis sessions.^33^

Among the few targeted network interventions that use in-degree centrality, Kim *et al.* in 2015 published a study examining targeted social network interventions to improve nutrition in villages across rural Honduras, with targeting on the basis of in-degree centrality as one possible method. They noted that targeted network interventions were successful in leveraging the intrinsic properties of social networks, namely our tendency to interact and form relationships with popular individuals.^10^ Targeted interventions also allowed for scalability and increased efficiency as information, and resources were directed toward a subset of individuals and subsequently disseminated to the rest of the network.^10^

Other targeted interventions have had success in leveraging social networks to promote KTs in randomized controlled trials (RCTs). An RCT by Sullivan *et al.* in 2012 trained KT recipients (navigators) across 23 Ohio hemodialysis facilities to provide tailored information and assistance in completing KT steps. They found that patients who interacted with navigators completed twice as many KT steps compared with controls.^18^ Another RCT by Patzer *et al.* in 2017 provided transplant education and engagement activities targeting hemodialysis clinic patients and staff across 134 hemodialysis clinics in Georgia, which increased referral rates for transplant, particularly among African American patients, thus improving equity in KT referral.^34^ Our findings, in conjunction with the literature demonstrating that information and behaviors are more easily transmitted between peers than from healthcare providers, are favorable for future social network interventions targeting influential patients on hemodialysis.^18,35^

### Limitations

This is a cross-sectional study with data from two geographically and demographically different hemodialysis facilities. There is a notable size disparity between the two facilities, which may have influenced our findings, as the number of potential ties formed is constrained to the overall size of the social network. Thus, larger networks have the potential for members to have greater in-degree centrality than smaller networks. Our surveys relied on self-reported patient data, so there may be discrepancies for variables, such as the length of time spent on hemodialysis and the number of transplant steps completed due to recall bias. It is possible that other unmeasured factors, such as frailty or family norms, may influence transplant attitudes. Finally, patients who did not participate in the study or patients who were excluded, such as the few participants who did not speak English, may have different attitudes toward KT and be less likely to participate in the clinic social network.

In this cross-sectional network study, influential patients at facility 1, as measured by in-degree centrality, had the greatest knowledge of successful KTs, positive attitudes toward transplant, and completed more steps toward transplant. Conversely, at facility 2, influential patients knew the least about successful KTs and had less positive attitudes and behaviors toward transplant. This has future implications for the implementation of network interventions targeting influential patients to promote transplantation and reduce disparities among patients with ESKD.

## Supplementary Material

## Data Availability

All data are included in the manuscript and/or supporting information.

## Supplemental Material

Supplemental Figure 1. Hemodialysis clinic enrollment scheme.

Supplemental Figure 2. Exponential random graph model goodness of fit.

Supplemental Figure 3. Exponential random graph model Markov Chain Monte Carlo.

Supplemental Table 1. Exponential random graph model by facility all variables.

Supplemental Figure 4. Age and sex between social network participants and total patients.
