# Supplementary material for: A Social Network Analysis of Hemodialysis Clinics: Attitudes Toward Living Donor Kidney Transplant among Influential Patients
Source: Kidney360. 2024 Feb 7;5(4):577–88. doi: 10.34067/KID.0000000000000383 (PMC11093547; doi:10.34067/KID.0000000000000383)
Supplement: Supplementary file 1 [file kidney360-5-577-s001.pdf]

Supplemental Material Table of Contents

Figure S1: Hemodialysis Clinic Enrollment Scheme 2
Figure S2: Exponential Random Graph Model Goodness of Fit 3
Figure S3: Exponential Random Graph Model Markov Chain Monte Carlo 4
Table S1: Exponential Random Graph Model by Facility All Variables 5
Figure S4: Age and Sex Between Social Network Participants and Total Patients 6

Figure S1: Hemodialysis Clinic Enrollment Scheme

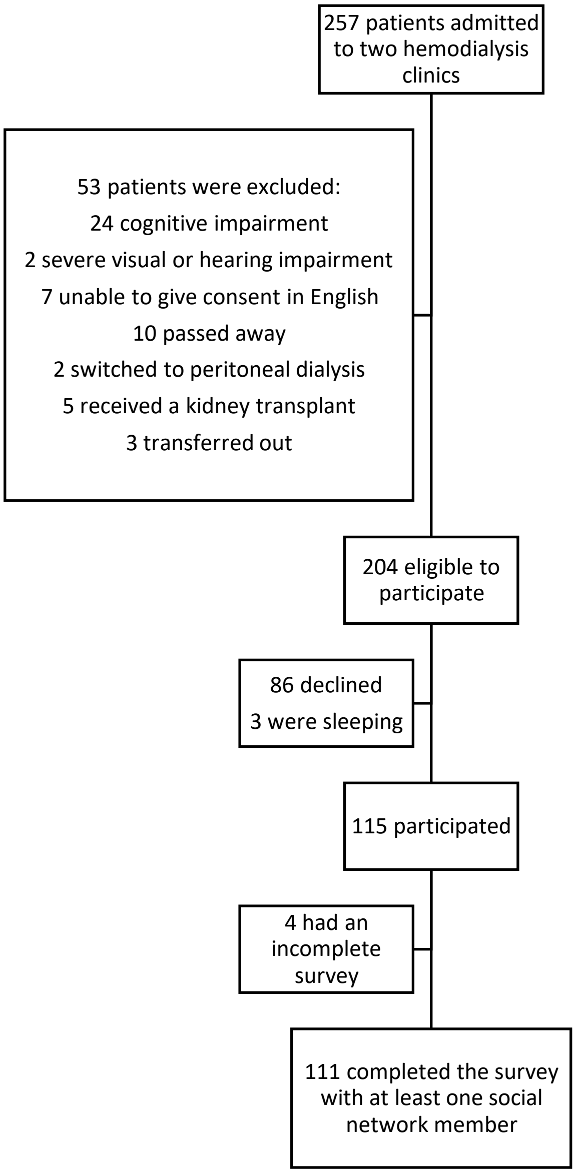

Figure S2: Exponential Random Graph Model Goodness of Fit

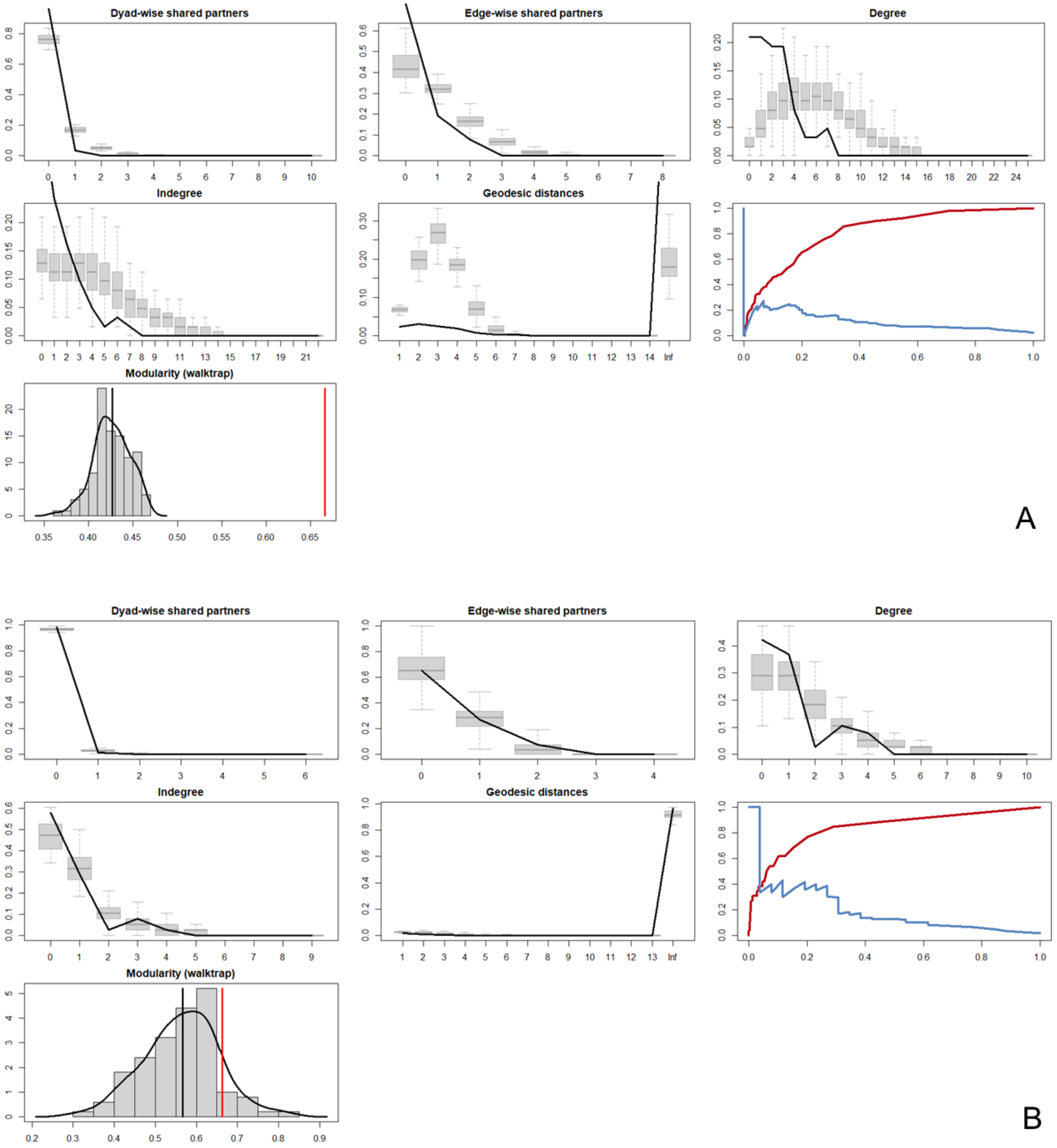

(A) Facility 1. (B) Facility 2. Goodness of fit was assessed by comparing our networks (black lines) to simulated networks (box plots), with overlap between the observed and simulated values indicating good fit. Receiver operating curves were generated to measure the relative frequency of true-positive predictions and false-positive predictions. Models for Facility 1 and Facility 2 demonstrated good fit with more true-positive predictions than false-positive ones. High modularity at both facilities was indicative of densely connected nodes.

Figure S3: Exponential Random Graph Model Markov Chain Monte Carlo

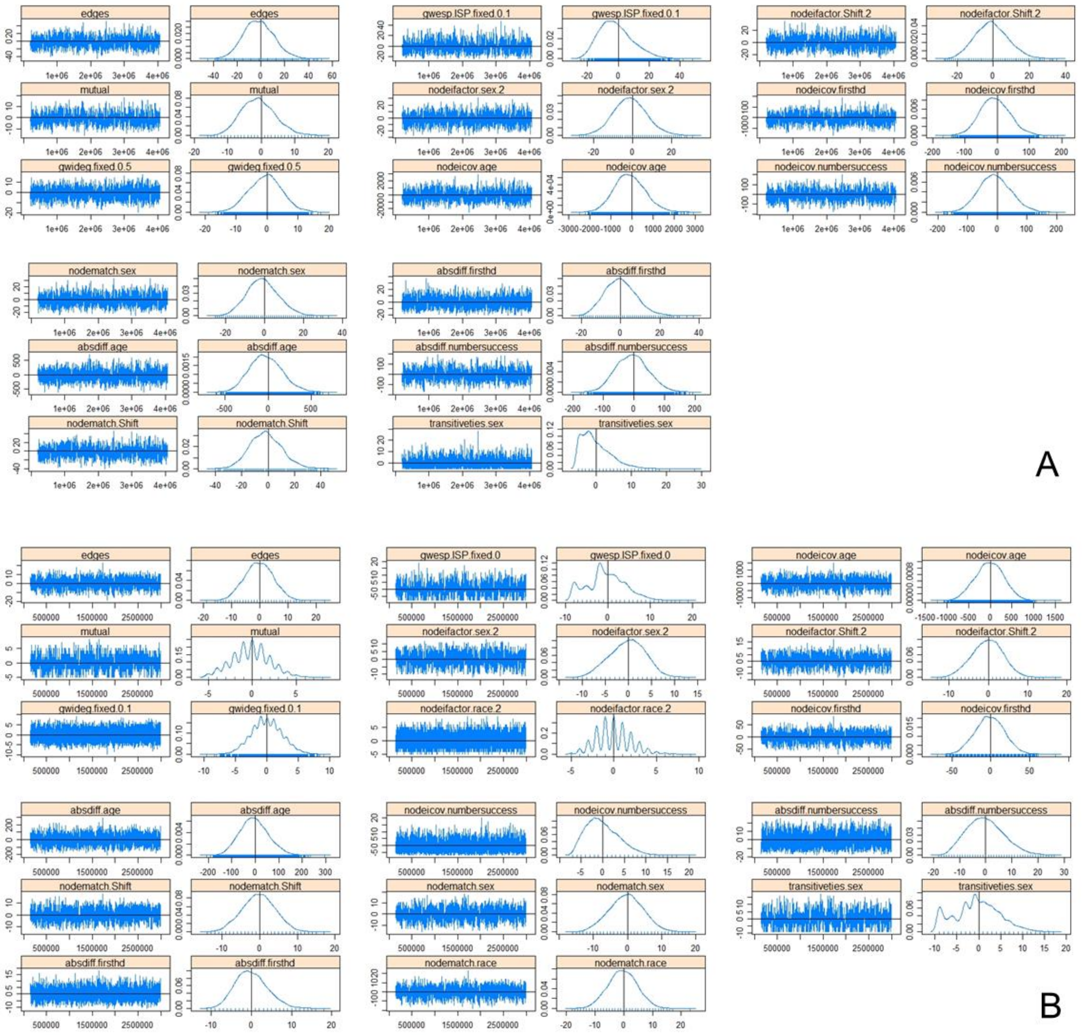

(A) Facility 1. (B) Facility 2. gwideg = geometrically weighted in-degree. ISP = incoming shared partner. nodeifactor = in-degree sociality (categorical variable). nodeicov = in-degree sociality (continuous variable). nodematch = homophily (categorical variable). absdiff = homophily (continuous variable). Markov Chain Monte Carlo diagnostics were generated to assess degeneracy and convergence of the models. The trace plots at both Facility 1 and Facility 2 indicated that the samples were a good approximation of the target distributions and the bell curves demonstrated convergence of both models.

Table S1: Exponential Random Graph Model by Facility All Variables

Variables	Facility 1	Facility 2
**Structural variables, *β* (SEM)**		
Edges	-4.07 (1.37)**	-6.85 (3.94)
Mutual	2.92 (0.41)***	2.96 (0.82)***
GWIDegree (0.5)	-0.63 (0.53)	
GWESP ISP (0.3)	0.30 (0.19)	
GWIDegree (0.1)		1.56 (0.98)
GWESP ISP (0)		0.83 (0.39)*
**Demographic variables, *β* (SEM)**		
Shift (sociality), REF = AM	0.31 (0.21)	-1.46 (0.79)
Shift (homophily)	1.88 (0.35)***	0.10 (0.43)
Age (sociality)	-0.03 (0.01)*	-0.03 (0.03)
Age (homophily)	-0.02 (0.01)	-0.05 (0.02)*
Race (sociality), REF = African American		-1.61 (0.86)
Race (homophily)		1.82 (0.73)*
Sex (sociality), REF = Male		1.15 (0.66)
Sex (homophily)	0.45 (0.25)	0.69 (0.42)
Transitivity (sex)	-0.45 (0.33)	
**Clinical variables, *β* (SEM)**		
Time on dialysis (sociality)	0.37 (0.26)	0.69 (0.73)
Time on dialysis (homophily)	-0.47 (0.19)*	-0.40 (0.36)
Number known successful kidney transplant (sociality)	0.11 (0.05)*	-1.09 (0.46)*
Number known successful kidney transplant (homophily)	-0.02 (0.04)	0.12 (0.22)
Importance of kidney transplant (sociality)	-0.13 (0.14)	0.27 (0.29)
Importance of kidney transplant (homophily)	-0.09 (0.10)	0.06 (0.14)
Trust doctors (sociality)	-0.24 (0.21)	0.14 (0.50)
Trust doctors (homophily)	0.12 (0.15)	-0.28 (0.32)
Highest Stage 1 (sociality), REF = Stage 0	0.06 (0.38)	-0.18 (1.03)
Highest Stage 2 (sociality), REF = Stage 0	0.24 (0.41)	1.36 (1.25)
Highest stage (homophily)	0.02 (0.21)	0.15 (0.51)
**Model factors**		
AIC	555.98	207.23
BIC	672.87	327.95
Log Likelihood	-256.99	-80.62

GWIDEGREE = geometrically weighted in-degree. GWESP = geometrically weighted edgewise shared partner. ISP = Incoming Shared Partner. REF = reference. AIC = Akaike's Information Criterion. BIC = Bayesian Information Criterion. ***p-value <0.001; **p-value <0.01; *p-value <0.05.

Figure S4: Age and Sex Between Social Network Participants and Total Patients

	Social Network Participants at Both Facilities (N = 111)	Total Patients at Both Facilities (N = 257)	*p* value
Age (mean ± standard deviation)	60 ± 13	62 ± 14	0.20
Female Sex (%)	48%	44%	0.50
